# Exploring Leptospiral proteomes to identify potential candidates for vaccine design against Leptospirosis using an immunoinformatics approach

**DOI:** 10.1038/s41598-018-25281-3

**Published:** 2018-05-02

**Authors:** Kumari Snehkant Lata, Swapnil Kumar, Vibhisha Vaghasia, Priyanka Sharma, Shivarudrappa B. Bhairappanvar, Subhash Soni, Jayashankar Das

**Affiliations:** 1 Gujarat Institute of Bioinformatics, Gujarat State Biotechnology Mission, Department of Science & Technology, Government of Gujarat, Gandhinagar, 382011 India; 2 Gujarat State Biotechnology Mission, Department of Science & Technology, Government of Gujarat, Gandhinagar, 382011 India; 3 Present Address: Gujarat Biotechnology Research Centre (GBRC), Department of Science & Technology, Government of Gujarat, Gandhinagar, 382011 India

## Abstract

Leptospirosis is the most widespread zoonotic disease, estimated to cause severe infection in more than one million people each year, particularly in developing countries of tropical areas. Several factors such as variable and nonspecific clinical manifestation, existence of large number of serovars and asymptomatic hosts spreading infection, poor sanitation and lack of an effective vaccine make prophylaxis difficult. Consequently, there is an urgent need to develop an effective vaccine to halt its spread all over the world. In this study, an immunoinformatics approach was employed to identify the most vital and effective immunogenic protein from the proteome of *Leptospira interrogans* serovar Copenhageni strain L1-130 that may be suitable to stimulate a significant immune response aiding in the development of peptide vaccine against leptospirosis. Both B-cell and T-cell (Helper T-lymphocyte (HTL) and cytotoxic T lymphocyte (CTL)) epitopes were predicted for the conserved and most immunogenic outer membrane lipoprotein. Further, the binding interaction of CTL epitopes with Major Histocompatibility Complex class I (MHC-I) was evaluated using docking techniques. A Molecular Dynamics Simulation study was also performed to evaluate the stability of the resulting epitope-MHC-I complexes. Overall, this study provides novel vaccine candidates and may prompt further development of vaccines against leptospirosis.

## Introduction

Leptospirosis is the most widespread zoonosis in the world and emerging as a major public health concern^1,2^. The global incidence of this tropical disease has been estimated over 1 million cases of severe infection in human amounting to nearly 60,000 deaths annually^3^. It is caused by pathogenic species of *Leptospira* and can get transmitted to human by direct contact with reservoir hosts or via exposure to surface water or soil contaminated with their urine^2,4^. Both wild and domestic animals can serve as reservoir hosts of *Leptospira*; however, animals such as rodents, pigs, cows, dogs and horses are the most common hosts and sources of infection to humans. Leptospirosis is predominantly an occupational disease where agricultural workers, veterinarians and mineworkers are mainly at risk because of their exposure to contaminated water, soil and infected animals during their regular activities^5^. The clinical symptoms of leptospirosis in humans are diverse, ranging from mild fever, chills, flu-like illness, headache, muscle aches, to acute disease form known as Weil’s syndrome^6^. The acute form is characterized by multiple organ complications, including acute renal and hepatic failure, cardiovascular collapse, jaundice, meningitis, pneumonitis and pulmonary haemorrhage, which in turn can lead to death^7,8^. Also, the disease has a major economic impact on the agricultural industry and companion animals, since it affects the livestock inducing abortions, infertility, stillbirths, reduced milk production and death, especially in developing countries^8,9^.

Presently, lack of proper therapeutics and vaccine against leptospirosis is increasing the burden of this disease day by day globally. The vaccination against leptospirosis in human populations may prove to be the most feasible approach for controlling the disease. Although, for over 100 years, whole cell inactivated and attenuated vaccines have been used for agricultural and companion animals and in some countries, also being used in human populations. But, due to their adverse effects, short-term immunity and insufficiency in inducing cross-protection, they have not been implemented globally^10,11^. *Leptospira* comprises more than 250 antigenically distinct serovars among pathogenic species^12,13^. This antigenic diversity of pathogenic *Leptospira* species makes up a challenge for the researchers to develop effective and cross-reactive vaccines.

In last two decades, classical research approach is being used to identify protein targets towards the development of subunit and recombinant vaccines against leptospirosis^14^. The current research for developing recombinant and subunit vaccines are mostly focused on leptospiral motility, outer-membrane proteins (OMPs), lipoproteins, lipopolysaccharides (LPSs) and virulence factors^15^. These proteins have been recognized as playing major role in the interaction of pathogens with host cells and possibly associated with pathogenesis; hence, is the major focus of current vaccine research. Among these, significant protection in the hamster model has been reported with several outer membrane proteins, including LipL32 and the leptospiral immunoglobulin-like proteins (Lig)^16–18^. However, protective efficacy of these candidates was limited and also had failed to induce cross-protection and sterile immunity. Therefore, a highly conserved target that can stimulate both humoral and cell mediated immunity against leptospirosis is crucial for the development of an effective vaccine. The current status of leptospiral vaccine development demonstrates that there is an urgent need for the discovery of new effective vaccine candidates to provide immunity against majority of serovars^18^.

The availability of omics and immunological data, and advances in the computational algorithms have improved the efficiency of vaccine development process by accelerating the research towards the identification of dominant immunogen and thereby potential epitope candidates^19–21^. Various studies have shown that epitope-driven vaccines could effectively stimulate protective immune responses against diverse pathogens, such as influenza virus, human immunodeficiency virus, hepatitis B virus, and hepatitis C virus^22–24^. As a matter of fact, identification of B-cell and T-cell epitopes is a crucial and noteworthy step for the epitope-based vaccine development. Immunoinformatics is now becoming ubiquitous in the field of vaccine development which utilizes genome and proteome based information and offers high level of confidence for the prediction of potential vaccine candidates^25^. Recently, the approach has been widely accepted for screening the effective immunogens for potential vaccine design of infectious diseases.

In the current study, with the help of immunoinformatics approach, whole proteome of *Leptospira interrogans* serovar Copenhageni strain L1-130 (LIC) was screened for the most immunogenic and conserved outer membrane (OM) proteins. Subsequently, various B-cell and T-cell epitopes were obtained that could induce protective humoral and cellular immune responses and may be characterized as effective vaccine candidates. Identifying the binding interaction between epitope and major histocompatibility complex (MHC) molecules is considered as the first step to vaccine design, as T-cell immunogenicity is correlated with the binding strength of epitopes and MHC molecule^26^. Therefore, these predicted epitopes were modelled and docked with MHC class I molecule and later on, their post-docking interaction analysis helped in the selection of optimal candidates for the development of peptide vaccines against leptospirosis.

## Results

This study aims to identify a cross-reactive and conserved potential vaccine candidate with the help of a comprehensive bioinformatics approach. *In silico* approach may prove as a beneficial and directive approach, whereas conventional methods focus more on pathogen cultivation and protein extraction, where testing of these proteins on a large scale is expensive and time-consuming^27,28^. Several *in silico* vaccine candidates have been reported by researchers which were known to produce promising preclinical and clinical trial results^29,30^.

In the present study, putative antigenic protein has been identified, of which B-cell (linear and conformational) and T-cell epitopes (HTL and CTL) have been predicted for the designing of peptide vaccines against leptospirosis.

### Identifying the highest antigenic protein

The selection of optimal immunogen is the first step for vaccine design; hence, to identify the most probable antigenic protein, the whole proteome of LIC constituting a total of 3654 proteins was analysed using VaxiJen v2.0 server. An overall score depicting antigenicity for each protein sequence was evaluated which indicated their potentiality to induce immune response; from which, 21 proteins having highest antigenicity score (>1.0) were selected for further analysis (Supplementary Dataset Table S1).

### Identification of Outer Membrane Protein (OMP)

It is generally envisaged that subcellular localization of a protein plays a vital role in determining its functionality. In Gram-negative bacteria, OMPs have diverse functions and were known to be involved in the interaction between bacterial cells and their host^31^. Moreover, in pathogenic bacteria, OMPs are proven to be the most promising vaccine candidates, due to its interaction with the host immune cells^32^; and hence, identification of OMPs are crucial for a reliable and rapid development of vaccine. Our analysis identified the subcellular localization of all 21 highly antigenic proteins as mentioned in the methods section, from which two proteins with UniProt ID: Q75FL0 and Q72PD2 were predicted to be OMP (Supplementary Table S2). Of these, protein Q72PD2 was uncharacterised and hence not considered for further analysis. Protein Q75FL0 has been annotated as lipoprotein and located in the outer membrane of LIC; therefore, selected as a candidate immunogen to accomplish the epitope based vaccine design.

### Primary and secondary structure determination

**Figure 1:**
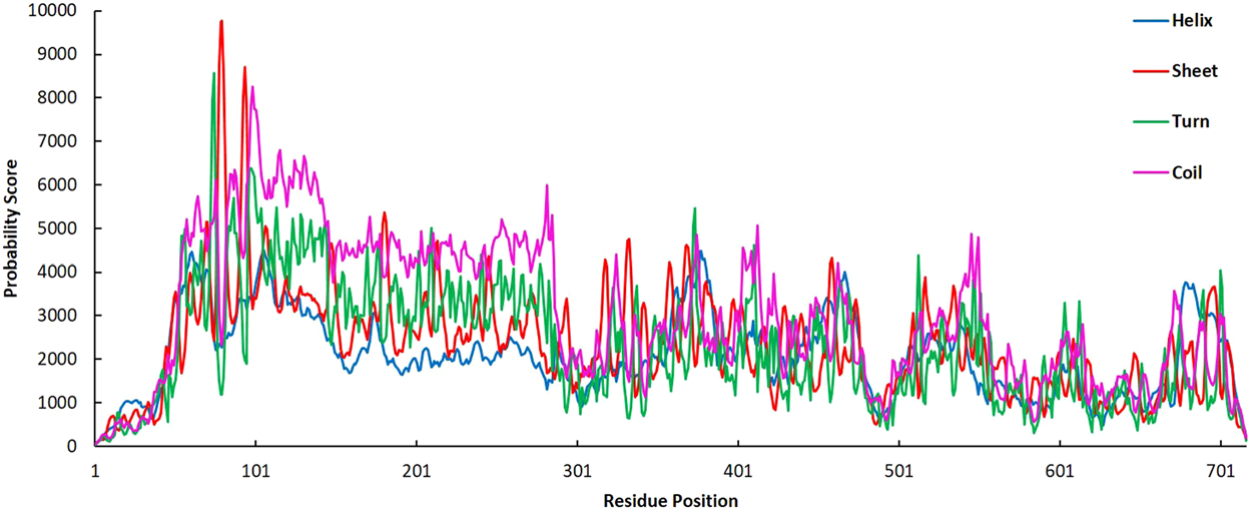
The probability graph of occurrence of helix, sheet, turn and coil in the secondary structure of the antigenic lipoprotein Q75FL0 of LIC. Here, the graph indicates the values of probability for each secondary structure at all amino acid positions of the protein.

Q75FL0, the most probable antigenic protein was analysed for its physicochemical properties and secondary structural characteristics. The results revealed the total length of protein as 717 amino acids with molecular weight of 73950.78 Daltons and theoretical Isoelectric point (PI) of 5.39 (Table 1). The instability index (II) was computed to be 32.21, which implies that the sequence of protein is stable. The sequence has about 59 negatively charged residues (Aspartic acid + Glutamic acid) and 48 positively charged residues (Arginine + Lysine). The amino-acid composition revealed that the protein has 10,261 atoms comprising Carbon (3181), Hydrogen (5058), Nitrogen (896), Oxygen (1121) and sulphur (5). The aliphatic index was calculated as 77.22. The grand average of hydropathicity (GRAVY) was calculated to be negative (−0.328). This negative value indicates the hydrophilic nature of protein and most of the residues to be located on the surface; hence this protein tends to have better interaction with other proteins. The secondary structure analysis of protein revealed that the protein is dominated by random coils (55.09%) followed by extended strand (23.15%), alpha helix (12.69%) and beta turns (9.07%). The calculated secondary structure parameters are shown in Table 2 and a plot for each residue position versus its probability score for being in helix, strand, turn and coil in Fig. 1.

**Table 1:** Different physico-chemical properties of lipoprotein Q75FL0 of LIC.

Criteria	Assessment
Number of amino acids	717
Molecular weight	73950.78
Formula	C_3181_H_5058_N_896_O_1121_S_5_
Theoretical pI	5.39
Total number of negatively charged residues (Asp + Glu)	59
Total number of positively charged residues (Arg + Lys)	48
Ext. coefficient	54780
Estimated half-life:	30 hours (mammalian reticulocytes, *in vitro*)
Instability index	32.21 (classified as stable)
Aliphatic index	77.22
Grand average of hydropathicity (GRAVY)	−0.328

**Table 2:** Secondary structure analysis of lipoprotein Q75FL0 through SOPMA.

Secondary Structure	Percentage
Alpha helix (Hh)	12.69
Extended strand (Ee)	23.15
Beta turn (Tt)	9.07
Random coil (Cc)	55.09
310 helix (Gg)	0.00
π helix (Ii)	0.00
Beta bridge (Bb)	0.00
Bend (Ss)	0.00

### Homology modelling and tertiary structure refinement

**Figure 2:**
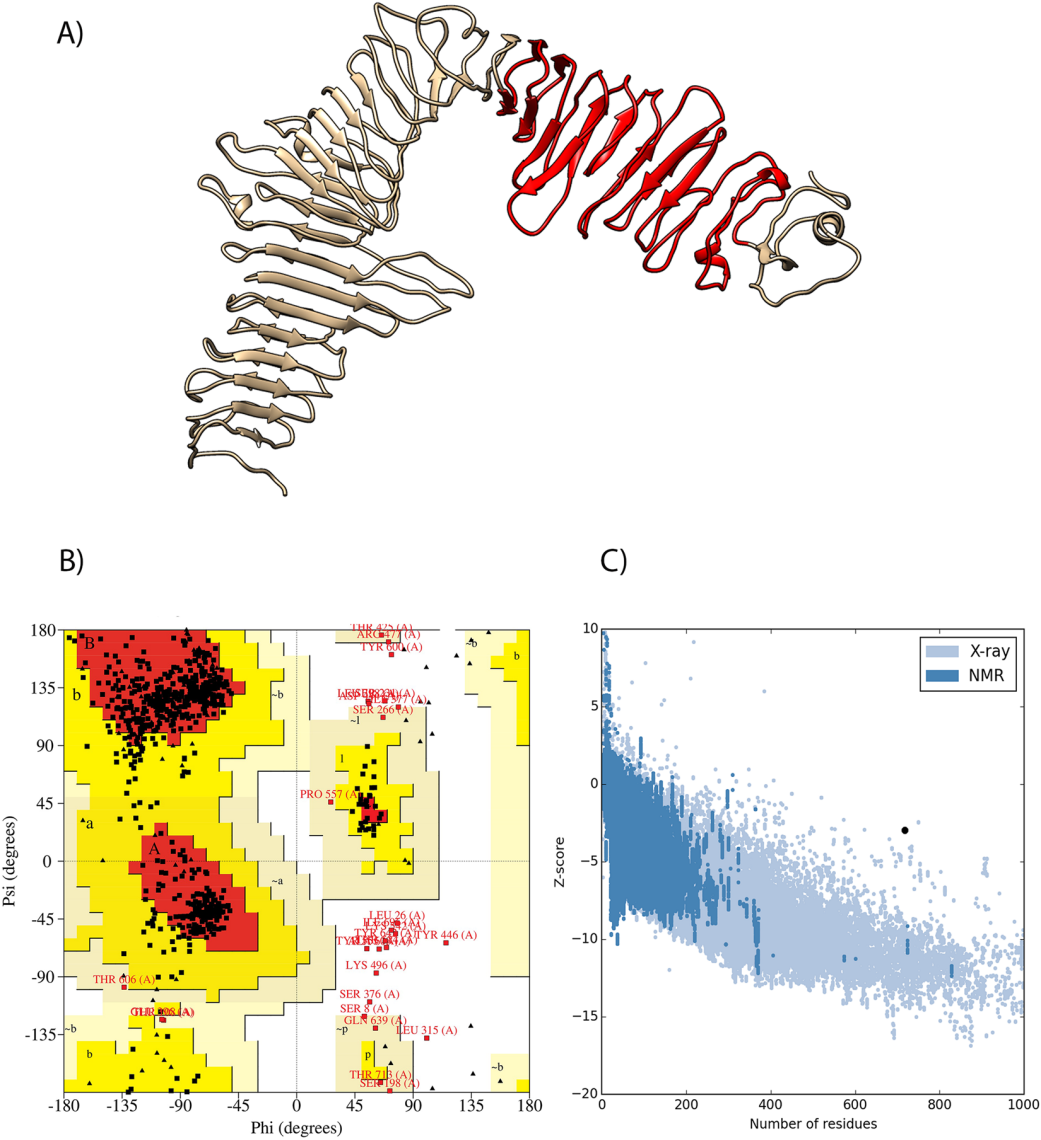
Predicted three-dimensional structure and validation of lipoprotein Q75FL0. (**A**) Three dimensional structure of the predicted model with the domain (LurC domain shown in red colour). (**B**) Ramachandran plot of refined 3D model showing 80.3%, 15.4%, 1.8% and 2.5% residues in the most favoured, additional allowed, generously allowed and disallowed region respectively. (**C**) Z-Score plot for 3D structure of model showing −2.97.

Based upon iterative threading assembly and simulation method, I-TASSER server^33^ generated five 3D models for the protein sequence and ranked all the model based on their C-scores. C-score values measure similarity between the query and template based on the significance of threading template alignment and the query coverage parameters. Typically, C-score values lie in between [−5 to 2], where a higher value denotes a model with a higher confidence and correct topology. The top ranked 3D model yielded C-score value of 0.16 which indicates that the model is having a good topology. The structure of the top ranked model with its functional domain (LurC domain: 439 − 661) is shown in Fig. 2(A). In addition to C-score, I-TASSER predicted up to ten closest structures in PDB and ranked them on the basis of TM-score and the root mean square deviation (RMSD) of atomic positions related to the best template used for 3D modelling. The closest protein structure and quality assessment parameters for the modelled structure are shown in Table 3. The top ranked model was refined using GalaxyRefine server^34^ and generated five refined models. Of these, the top ranked structure was selected on the basis of Ramachandran plot (80.3% in favoured region). Consequently, the quality of refined model was evaluated by using PROCHECK, ProSA-Web and ModFold6. PROCHECK calculates the steriochemical quality of the protein and depicted Ramachandran plot as shown in Fig. 2(B). The Ramachandran plot analysis of refined structure revealed that 80.3% of residues were located in most favoured region followed by 15.4% in allowed and 1.8% in generously allowed region, while only 2.5% of the residues were in disallowed region. However, ProSA-Web calculated Z-score of −2.97 indicating the model was not in the range of native protein conformation (Fig. 2(C)). Furthermore, the ModFold6 sever was used to evaluate the overall quality of the model (Table 3).

**Table 3:** Protein structurally close to the model in the PDB and Quality assessment of the model.

Closest structure in PDB	C-score	TM-score	RMSD	Quality assessment			
				Ramachandran most favoured residues (%)	Z-Score	ModFold score	Confidence and *p-*value ModFold
5n8pA	0.16	0.971	0.90	80.3	−2.97	0.2560	POOR: 4.628E-1

C-score of model indicates the global topology, higher score means the better model (>−1.5 considered as a good topology). TM-score meas0075res the significance of the structural alignment between modelled protein and validated structures in PDB. RMSD: the root mean square deviation (RMSD) between residues that are structurally aligned.

### Identification of linear B-cell epitopes

The identification and characterization of B-cell epitopes in target antigen is a key step in the epitope-based vaccine design. The Kolaskar and Tongaonkar’s method^35^ of the Immune Epitope Database (IEDB) Analysis Resource predicts the antigenic peptides by analysing the physicochemical properties of amino acid residues and their abundance in experimentally determined antigenic epitopes. The result revealed that the protein sequence of 717 aa has 26 antigenic peptides falling in the range of 6-22 amino acids length (Table 4). In addition, maximum residual score for each amino acid residue was also predicted. Out of 717 amino acids, 343 amino acids have residual score ≥ 1.008. Proline and Valine at the 400^th^ and 401^st^ positions, found in the antigenic peptide ^**394**^KYEVLL**PV**AAVPT^**406**^, was identified as having the highest antigenic residual score of 1.208. It should be noted that the epitopes ^**20**^MKKILILLIALSFAVFGCSHK^**40**^ and ^**42**^KGILLPFLTLLNQ^**54**^ were recognized as allergic to human; henceforward, they could not be considered as vaccine candidates. Fortunately, within ^**617**^WA**ILVPGA**^**624**^ and ^**5**^**YSSSFILII**KKG^**16**^ epitopes, some residues were also predicted as conformational as well as CTL epitopes, so can be considered as good candidates for peptide vaccine design. Moreover, the result indicated that the average antigenic propensity score of the predicted epitope was 1.008 while the minimum and maximum score was 0.855 and 1.208 respectively. The graphical representation of predicted antigenic residues based on the sequence position (X-axis) and antigenic propensity (y-axis) are shown in Supplementary Fig. S1 (Supporting Information). The detailed information of predicted epitopes including their conservancy and allergenicity are shown in Table 4.

**Table 4:** Antigenic linear B-cell epitopes of lipoprotein Q75FL0 with their conservancy and allergenicity.

No.	Start position	End position	Peptide	Peptide Length	Allergenicity	Conservany (%)
1	5	16	**YSSSFILII**KKG	12	No	2.17
2	20	40	MKKILILLIALSFAVFGCSHK	21	Yes	30.43
3	42	54	KGILLPFLTLLNQ	13	Yes	60.87
4	76	82	GSVVIVD	7	No	76.09
5	91	96	SGVLLP	6	No	41.30
6	301	321	**GSIPFTY**NTVQTIPLNLVVTD	21	No	54.35
7	329	335	GATVIVS	7	No	93.48
8	340	345	HILFQG	6	No	89.13
9	359	371	VETALGQITLEIT	13	No	65.22
10	377	390	ISNVINLINVVGIN	14	No	54.35
11	394	406	KYEVLLPVAAVPT	13	No	17.39
12	427	432	SSLIRI	6	No	71.74
13	434	449	AEGV**STVAYEDLY**PSA	16	No	73.91
14	455	462	NDYVLHIH	8	No	97.83
15	470	477	AGDVIRLR	8	No	97.83
16	479	486	TYQHVARG	8	No	91.30
17	490	504	KHTFFLKLPVAVGAT	15	No	60.87
18	506	512	SRKVVRE	7	No	84.78
19	522	529	**TKTV**SSSD	8	No	63.04
20	551	570	PGGVYKPGYIATIEIVFNSP	20	No	56.52
21	576	588	**LGSYPY**DIFIKVI	13	No	60.87
22	594	600	IHFPGLY	7	No	100.00
23	617	624	WA**ILVPGA**	8	No	95.65
24	666	674	QAKVFPVPD	9	No	95.65
25	680	686	MGFLLRS	7	No	58.70
26	691	712	AILIAILLIGAGAAVAYILKRR	22	No	56.52

26 antigenic sites were predicted. Residues underlined and in bold were also predicted as conformational B-cell and CTL epitopes respectively.

Since potential B-cell epitopes have several key features, including surface accessibility, fragment flexibility and hydrophilicity which are crucial for predicting B-cell epitopes, these were analysed by different methods implemented in IEDB. The surface accessibility prediction showed that the maximum surface probability value of predicted peptides was calculated as 5.282 at amino acid residues from 320 to 325 with the sequence of hexapeptide ^**320**^TDK**Q**SK^**325**^, where 323Q is a surface residue, while the minimum surface probability score was 0.043 for the peptides ^**23**^IL**I**LLI^**28**^, where 25I is the surface residue. Peptides with threshold value > 1.0 have high probability to be located on the surface^36^. The Graphical representation of predicted surface accessible residues on the basis of their sequence position (x-axis) and surface probability (y-axis) are shown in Supplementary Fig. S2 (Supporting Information).

Surface flexibility of peptides is also an important feature for predicting antigenic peptides, as experimental data have shown that the antigenic regions of peptide that interact with antibody are probably more flexible and also well suited for choosing cross-reacting peptide^37^. Based upon the temperature factor or B factor of Cα atom, Karplus and Schulz flexibility method of IEDB predicted the flexible regions on the protein. The analysis showed that the maximum flexibility value was 1.166 at amino acid position 164 to 170 with a sequence of GSSSSSG, while the minimum flexibility score was 0.891 for the peptide ^703^AAVAYIL^709^. Peptides with low B-factor value are predicted to have well-organized structure. The result of predicted surface flexible regions is shown in Supplementary Fig. S3 (Supporting Information).

The Parker hydrophilicity scale method^38^ was employed to identify the hydrophilic peptides in the protein sequence as discussed in the method section. The maximum hydrophilicity score calculated by this method was 7.4 with a peptide sequence of ^406^TDTDKDG^412^; however, the minimum score was calculated as −7.243 for peptide sequence of ^24^LILLIAL^30^. The graphical representation of predicted hydrophilic residues on the basis of their sequence position (x-axis) and surface hydrophilicity (y-axis) are shown in Supplementary Fig. S4 (Supporting Information).

### Structure-based Epitope Prediction

**Figure 3:**
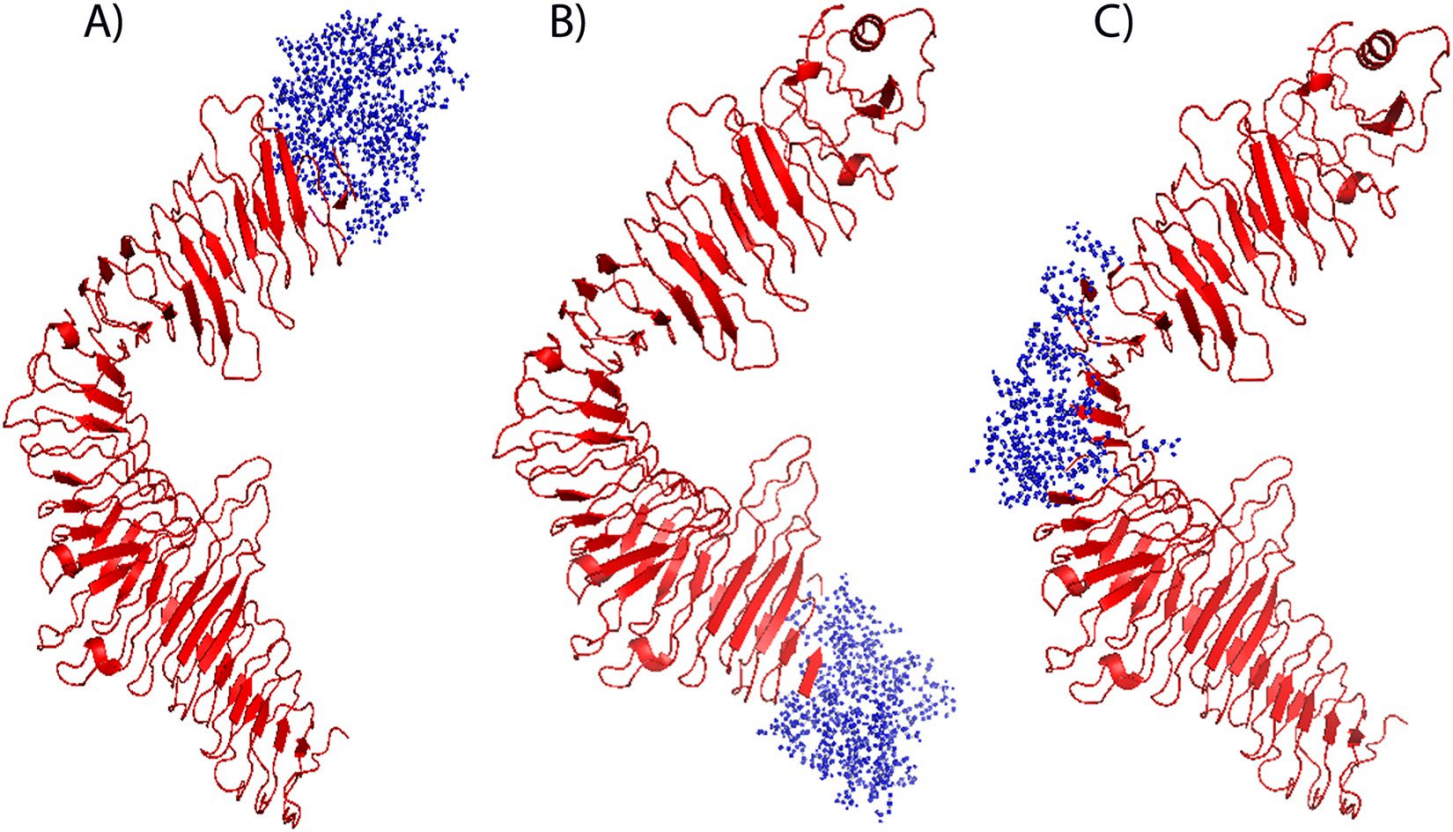
3D representation of conformational B-cell epitopes (**A**–**C**) of protein. The predicted epitope residues are denoted by blue colour (ball & stick), and the rest of the residues are in red (Cartoon).

In order to find conformational B-cell epitope in 3D structure, Ellipro^39^ was used. This tool predicts the epitopes based on the geometrical properties of the protein structure and it discriminates predicted epitopes from non-epitopes on the basis of known protein antibody complex. The conformational B-cell epitopes with a protrusion index (PI) value above 0.7 were selected. The score (PI) reflects the percentage of protein atoms that extend beyond the molecular bulk and are responsible for antibody binding^39^. The highest probability of a conformational epitope was computed as 85.5% (PI score: 0.855). The Amino acid residues present in conformational epitopes, the number of residues and their scores are depicted in Table 5, whereas the graphical representations are shown in Fig. 3.

**Table 5:** Predicted conformational B-cell epitopes of protein.

No.	Residues	Number of Residues	Score
1.	I619, V621, P622,G623, A624, W625, K626, Y627, P628, Y629, G631, L632, D633, R635, D637, A638, Q639, T640, G641, Y642, K643, E644, F645, W648, V649, A650, S651, N652, G653, T654, S655, Y656, K657, D658, W659, Y660, K661, H662, I663, T664, N665, Q666, A667, K668, V669, F670, P671, V672, P673, D674, E675, D676, S677, E678, L679, M680, G681, F682, L683, L684, R685, S686, M687, K688, K689, N690, A691, I692, L693, I694, A695, I696, L697, L698, I699, G700, A701, G702, A703, A704, V705, A706, Y707, I708, L709, K710, R711, R712, T713, L714, S715, Q716, A717	93	0.855
2	M1, N2, V3, E4, Y5, S6, S7, S8, F9, I10, L11, I12, I13, K14, K15, G16, Y17, R18, V19, M20, K21, K22, I23, L24, I25, L26, L27, I28, A29, L30, S31, F32, A33, V34, F35, G36, C37, S38, H39, K40, K41, K42, G43, I44, L45, L46, P47, F48, L49, T50, L51, L52, N53, Q54, D55, A56, G57, A58, S59, T60, A61, K62, S63, E64, S65, S66, T67, G68, T69, A70, T71, A72, S73, N74, G76, V78, V79, I80, V81, D82, N83, N88, N89, S90, S91, L95, P96	87	0.84
3	S347, N348, N349, I350, L363, G364, Q365, I366, T367, L368, E369, I370, T371, A373, N382, L383, I384, N385, V386, V387, G388, I389, N390, R391, E392, I393, K394, Y395, V404, P405, T406, D407, T408, D409, K410, D411, G412, V413, P414, D415, T416, L417, D418, R431, P433, A434, E435, G436, D456, L476, R477	51	0.738

Residues (underlined) were also predicted as antigenic residue of linear B-cell epitopes.

### Identification of Helper T Lymphocyte (HTL) cell epitopes

HTL is crucial for inducing and generating an efficient humoral or cytotoxic T-cell response; therefore, in order to find the peptides that may trigger the MHC-II restricted T-cell response, the NetMHCIIpan 3.1 server^40^ was utilised. Prediction was made for Human Leukocyte Antigen-DR (HLA-DR) alleles and only strong binder (SB) epitopes having IC_50_ value < 50 nM with high binding affinity to HLA-DR, were considered. As a result, a total of 33 SB T-cell epitopes for the query sequence were predicted and are shown in Supplementary Dataset Table S3. It has been known that the binding strength of HTL epitope to the HLA-DR is a key factor in immunogenicity of the T-cell epitope and a good T-cell epitope candidate should interact with maximum number of HLA alleles^41,42^. Therefore, based on the highest number of HLA-DR binding alleles, the top 10 epitopes were selected as putative HTLs (Table 6). Of these, epitope sequence ^17^YRVMKKILI^25^ interacting with highest number of HLA-DR alleles (336 alleles) can be considered as a good candidate for subunit-vaccine design. On the other hand, the peptide sequence ^48^FLTLLNQDA^56^ interacting with 120 alleles was predicted to be allergenic to human; hence, could not be considered for vaccine design. Moreover, the conservancy of all selected epitopes were found in the range of 2.17 to 95.65%, representing 46 serovars of pathogenic *Leptospira spp*. The epitope ^565^IVFNSPVKK^573^ interacting with 132 HLA-DR alleles was predicted to be at the highest conservancy level (*i*.*e*. conserved among 44 serovars). Details of predicted HTL epitopes along with their binding HLA-DR alleles are shown in Supplementary Dataset Table S3.

**Table 6:** Top 10 HTL epitope with their allergencity and conservancy, selected on the basis of maximum number of HLA-DR binding alleles.

S.No.	Peptide	Position	Total No of HLA-DRB binding Alleles	Allergenicity	Conservancy (%)
1	YRVMKKILI	17–25	336	No	2.17
2	AVAYILKRR	704–712	301	No	58.7
3	ILIIKKGYR	10–18	279	No	2.17
4	LIIKKGYRV	11–19	265	No	2.13
5	YILKRRTLS	707–715	259	No	89.13
6	AYILKRRTL	706–713	244	No	89.13
7	MGFLLRSMK	680–688	174	No	56.52
9	VAYILKRRT	705–713	148	No	58.7
8	IVFNSPVKK	565–573	132	No	95.65
10	FLTLLNQDA	48–56	120	Yes	60.87

### CTL Epitope prediction

Cytotoxic-T-lymphocytes (CTLs) are critically one of the vital instigators of cellular immunity and play an important role in eliminating the infected cells. Hence, to identify the potential T-cell epitope that is recognized by CD8+ T-cell and stimulate both long-lasting and exclusive cytotoxic immune response, NetCTL 1.2 server^43^ was employed. This server identifies the epitope candidates by using artificial neural network and calculates a combined score for a peptide sequence based on their MHC-I binding affinity, proteasomal C-terminal cleavage and TAP transport efficiency all together^43^. Herein, a total of 12 peptide sequences were predicted as CTL epitopes whose prediction scores were greater than 0.75 (Table 7). Of these, epitopes ^438^STVAYEDLY^446^, ^299^QIGSIPFTY^308^ and ^**619**^ILVPGAWKY^627^ have also been predicted to be antigenic and were conserved among 46 serovars of pathogenic species, which suggest that they could be promising vaccine candidates. In addition, they were predicted to have positive immunogenicity, wherein the positive score of immunogenicity signifies the high potentiality to stimulate strong CTL response. The peptide sequence ^526^SSSDLNLGI^534^ was predicted as antigenic for human, so cannot be considered for vaccine design. The details of predicted CTL epitopes with their IEDB immunogenicity score, conservancy value and allergenicity are shown in Table 7.

**Table 7:** Predicted CTL epitopes.

No.	Residue number	Peptide sequence	Predicted MHC binding affinity	Rescale binding affinity	C-terminal cleavage affinity	Transport affinity	Prediction score*	MHC ligand	Immunogenicity Using IEDB	Conservancy (%)	Allergenicity
1	438	**STVAYEDLY**	0.5117	2.1724	0.8673	3.081	2.4565	YES	0.15566	93.48	No
2	573	KTA**LGSYPY**	0.4167	1.7691	0.9023	3.128	2.0609	YES	−0.13079	45.65	No
3	648	WVASNGTSY	0.395	1.6771	0.8945	3.101	1.9663	YES	−0.19207	76.09	No
4	334	VSDNEGHIL	0.3292	1.3978	0.518	0.89	1.52	YES	0.23515	82.61	No
5	5	**YSSSFILII**	0.3231	1.3719	0.5421	0.384	1.4724	YES	0.08751	2.17	No
6	517	VTDLT**TKTV**	0.2346	0.996	0.9599	0.188	1.1493	YES	−0.08894	60.87	No
7	299	QI**GSIPFTY**	0.1839	0.7807	0.972	2.764	1.0647	YES	0.08517	91.30	No
8	619	**ILVPGA**WKY	0.175	0.7431	0.9744	3.199	1.0492	YES	0.13301	95.65	No
9	99	NSDSSSNAT	0.2342	0.9944	0.1875	−0.663	0.9893	YES	−0.4587	54.35	No
10	526	SSSDLNLGI	0.1834	0.7787	0.9216	0.671	0.9505	YES	−0.03783	56.52	YES
11	609	YLDSNNFPW	0.17	0.7219	0.8792	0.809	0.8942	YES	−0.07934	93.48	No
12	653	GTSYKDWYK	0.1626	0.6905	0.5908	0.401	0.7991	YES	−0.06176	78.26	No

Residues in Bold indicate that were also predicted as antigenic sites in linear B-cell epitopes.

### Molecular Docking of CTL-epitopes with HLA-A*0201

Molecular docking was performed to determine binding affinities between all the predicted CTL epitopes and HLA-A*0201 (as discussed in the methods section). Out of 12 predicted CTL epitopes, 11 CTL epitopes excluding the allergenic one *i*.*e*. SSSDLNLGI were docked to MHC class I HLA-A*0201. The analysis revealed that out of 11, only four predicted CTL epitopes (STVAYEDLY, ILVPGAWKY, QIGSIPFTY and KTALGSYPY) showed strong binding affinities in terms of global energy and attractive van der Waals energy (vdW) ranging from – 61.00 to − 48.99 kcal/mol and − 28.32 to − 23.35 kcal/mol respectively (Table 8 and Supplementary Table S4). Of these, three epitopes (STVAYEDLY, ILVPGAWKY and QIGSIPFTY) were found to contain antigenic amino acid residues, positive IEDB immunogenicity score and high degree of conservancy (Table 7). The presence of these properties can lead an epitope to be a promising peptide vaccine candidate. Moreover, seven epitopes (NSDSSSNAT, GTSYKDWYK, VSDNEGHIL, YSSSFILII, VTDLTTKTV, YLDSNNFPW and WVASNGTSY) have shown poor binding affinities in terms of global energy ranging from – 39.30 to – 15.27 kcal/mol as tabulated in the Supplementary Table S4. We found that the docking energies of aforementioned epitopes (lowest global energy –39.30 kcal/mol) are nowhere close to those of top three epitopes (STVAYEDLY, ILVPGAWKY and QIGSIPFTY; highest global energy –48.99 kcal/mol).

**Table 8:** CTL epitopes and HLA-A*0201 interactions.

Peptide	Global energy (kcal/mol)^a^	Attractive vdW energy (kcal/mol)	H-bond energy (kcal/mol)	H-bond interaction		
				Epitope-MHC atom pair^b^	Distance initial^c^ (Å)	Distance after MD^d^ (Å)
STVAYEDLY	−55.57	−23.35	−1.64	SER 1 N-GLU 53 OE2	3.16	nd
				SER 1 OG-GLU 53 OE1	2.45	nd
				SER 1 N-GLU 53 OE1	3.68	nd
				TYR 5 OH-ARG 181 NH2	3.22	nd
				TYR 9 OH-TRP 51 NE1	2.69	nd
				GLU 6 OE2-ARG 48 HH22	nd	2.31
				GLU 6 OE2-ARG 48 HH12	nd	1.73
				ASP 7 OD1-ARG 48 HE	nd	1.87
				ASP 7 OD1-ARG 48 HH11	nd	2.15
				ASP 7 OD2-ARG 48 HH11	nd	1.87
				SER 1 H3-GLU 53 OE1	nd	1.74
ILVPGAWKY	−56.90	−26.02	−3.82	LEU 2 N-TYR 99 OH	3.02	nd
				ILE 1 O-LYS 66 NZ	3.72	nd
				ILE 1 N-GLU 63 OE1	3.96	nd
				ILE 1 H3-GLU 63 OE1	nd	1.59
QIGSIPFTY	−48.99	−23.86	−1.41	GLN 1 N-ASP 30 O	2.12	nd
				THR 8 OG1-ASP 30 OD2	2.11	nd
				GLN 1 OE1- TYR 27 OH	2.86	nd

^a^FireDock energy for the best ranked complex.

^b^Interacting pair of atoms and residues between epitope and HLA-A*0201.

^c^Initial distance between the H-bond acceptor and the donor.

^d^Distance between the H-bond acceptor and the donor after the MD simulation. nd = H-bond not detected.

**Figure 4:**
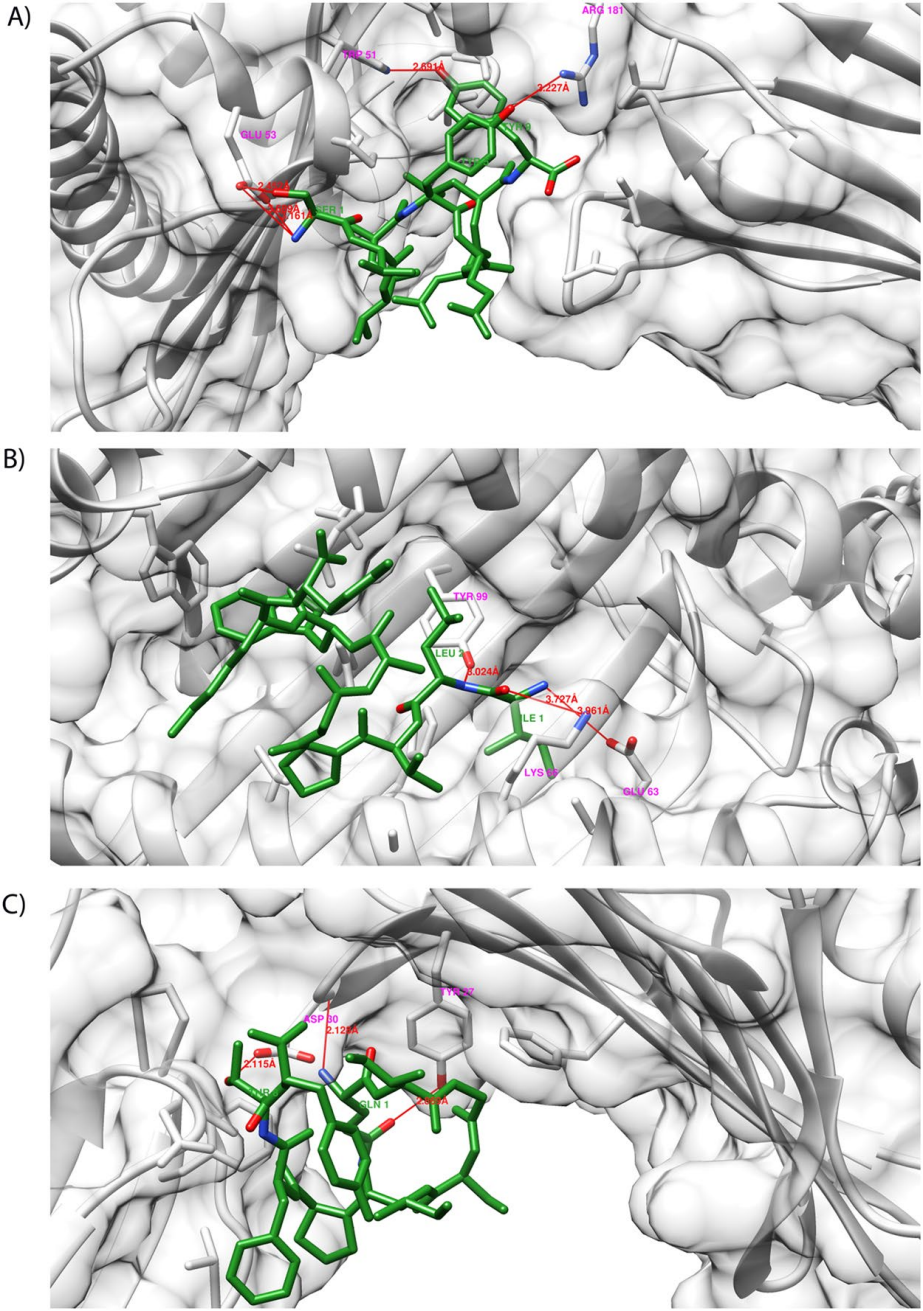
Molecular interaction analysis of predicted CTL epitopes docked to MHC-I molecule. (**A**) STVAYEDLY-HLA-A*0201 docking complex interacting with five hydrogen bonds; (**B**) ILVPGAWKY-HLA-A*0201 complex interacting with three hydrogen bond; (**C**) QIGSIPFTY-HLA-A*0201 complex making three hydrogen bonds. The residues forming H-bonds are labelled in magenta and green colour for HLA protein and epitope respectively. Each residue participating in H-bonding are coloured with Atom type (Red: Oxygen, Blue: Nitrogen).

Furthermore, the post-docking analysis result revealed the presence of five hydrogen bonds in STVAYEDLY**-**HLA-A*0201 complex within a distance of 3.68 Å; thus, pointing out the stability of the docked complex (the details are available in Table 8). Likewise, three hydrogen bonds were detected between ILVPGAWKY**-**HLA-A*0201, QIGSIPFTY**-**HLA-A*0201 and KTALGSYPY**-**HLA-A*0201 complex within a distance of 3.96, 2.869 and 3.85 Å respectively (Table 8 and Supplementary Table S4). Moreover, the reliability of the docked complex seems to be well-preserved by the formation of hydrogen bonds. Unfortunately, peptide STVAYEDLY, QIGSIPFTY and KTALGSYPY were not found to be binding in the binding groove of HLA-A*0201; while, ILVPGAWKY binds within the groove of HLA-A*0201. Overall analysis of the result showed that ILVPGAWKY epitope can be considered as a potential vaccine candidate for the epitope-driven vaccine design, as predicted to have lowest global energy and also binding within the groove of HLA-A*0201, which point out the stability of docked complex. In addition, this peptide was found to be conserved among 44 serovars of pathogenic *Leptospira*. Molecular interactions of the top three docked complexes with CTL-epitopes and HLA-A*0201 are shown in Fig. 4. The detailed analysis of other docked complexes is shown in supplementary Table S4. Further, the molecular interaction analysis of these predicted CTL epitopes (other eight CTL epitoes) docked to HLA-A*0201 protein are shown in supplementary Fig. S5. Also, the binding mode of each epitope to HLA-A*0201 proteins for all 11 complexes are shown in supplementary Fig. S6. Thus including KTALGSYPY, all epitopes having weaker binding were treated as negative controls and used for the stability analysis along with the top three epitopes meeting additional criteria.

### Molecular dynamics simulations

The stability of epitope-MHC I docked complexes was further studied by molecular dynamics (MD) simulation using GROMACS v2016.3. During MD simulation, all peptides moved from their original docking site and made new favourable interactions (top three epitopes are shown in Table 8 and other eight epitopes in Supplementary Table S4). While peptide STVAYEDLY retained its original docking conformation, others moved to adopt a slightly different, yet docked, conformations. Whereas YLDSNNFPW did not retain its original confirmation (Supplementary Fig. S7). The Root Mean Square Deviations (RMSDs) of top three docked complexes (ILVPGAWKY**-**HLA-A*0201, QIGSIPFTY**-**HLA-A*0201 and STVAYEDLY-HLA-A*0201) after simulation with respect to the complexes before simulation were 1.06, 1.36 and 0.94 Å respectively.

## Discussion

The global incidence of leptospirosis is increasing year by year, from an initial estimate of approximately 500,000 cases in 1999^44^, to over a million of severe cases in humans, resulting in ~60,000 fatalities in 2015^3^. To overcome this disease burden, there is an urgent need of improved preventive measures against the disease. Vaccination is one of the most effective means to efficiently, rapidly and affordably improve the public health and the most feasible way to eradicate this infectious disease. The search for effective vaccines to prevent leptospirosis has been on-going for many decades^15^. Despite this, the development of broadly effective vaccines against leptospirosis remains desirable and yet challenging task due to the wide array of antigenic diversity among pathogenic species^12^. The currently available vaccines against leptospirosis consist of whole-cell inactivated and formalin-killed leptospires (bacterin). However, these vaccines often show severe side-effects and are unable to stimulate cross-protection against different serovars and hence, their efficacy is limited. Therefore, current vaccine research is mostly focused on peptide and subunit vaccines as compared to whole organism vaccines because subunit vaccines contain specific immunogenic components of the pathogens responsible for the infection rather than the whole pathogen, which may result in severe side-effects. In *Leptospira*, vaccine targets include OMPs, lipoproteins and transmembrane proteins. Indeed, the most promising vaccine candidate so far described is the surface protein Lig, while OM LipL32 is the most studied leptospiral protein^15^. However, the efficacy of these vaccine candidates was limited and failed to induce cross-protective immunity. Therefore, the identification of other, more conserved, immunogenic OM proteins would be highly desirable for the development of cross-protective vaccine against leptospirosis. It is well-known that in *Leptospira*, OMPs exhibit high level of conservancy and are associated with pathogenesis; therefore, likely to be the most promising and successful candidates for peptide vaccines.

This study aims to screen and scrutinize the most antigenic OMP of the LIC, one of the most studied pathogenic *Leptospira* strains^14^, and to predict the possible antigenic B-cell and T-cell epitopes for epitope-based or peptide vaccine development by using *in silico* proteome wide-screening strategy. Several researchers have used *in silico* approach for identifying and designing of vaccine candidates^45–50^ and some of them achieved promising clinical trial results (for example ref.^30^). Screening using *in vitro* assays further reduces the number of vaccine candidates and hence, the number of laboratory animals required for efficacy testing. With immunoinformatics approaches, it is now feasible to screen the entire antigenic repertoire of a pathogen that could progress the discovery of potential vaccine candidates and may eventually improve existing vaccines. In our study, 21 proteins were predicted as highly immunogenic (antigenicity score > 1). Of these, two proteins (Q75FL0 and Q72PD2) were found to be located on the outer membrane. The Q72PD2 protein was annotated as hypothetical protein; hence, was excluded for further analysis. BLASTP analysis revealed that Q75FL0 is 100% identical to LruC domain-containing protein of LIC with 97% of query coverage and hence, may be characterised as LruC protein. The protein LruC was formerly described as leptospiral recurrent uveitis-associated protein C^51,52^. Experimentally, LruC protein was proven to be an OM lipoprotein and may have a role in pathogenesis of leptospiral Uveitis^52^. In addition, the LruC lipoprotein was found to be conserved among pathogenic *Leptospira* species. Thus, lipoprotein Q75FL0 could be the most promising new vaccine candidate for leptospirosis because of common and important features, including OM localization, conservation, and eliciting antibody production in patients^52,53^. Vaccination, or immunization works by stimulating antigen specific B-cells or CTLs and HTLs immune response. Consequently, B-cells, HTLs and CTLs epitope were predicted in the Q75FL0 lipoprotein. An effective peptide-based vaccine should contain both B-cell and T-cell epitopes to be able to elicit humoral and cellular immunity respectively. Several researchers have identified combined B-cell and T-cell epitopes of Leptospiral OMPs for diagnosis and vaccine purpose (see for example refs^54,55^). In our study, a number of peptides were predicted as comprising both B-cell and T-cell epitopes including ^5^**YSSSFILII**KKG^16^, ^**301**^**GSIPFTY**NTVQTIPLNLVVTD^321^, ^434^AEGV**STVAYEDLY**PSA^449^, ^522^**TKTV**SSSD^529^, ^576^**LGSYPY**DIFIKVI^588^ and ^617^WA**ILVPGA**^624^, thus could induce humoral as well as cell mediated immunity and hence, can be considered for the development of peptide vaccines against leptospirosis. Furthermore, surface accessibility, surface flexibility as well as hydrophilicity for the B-cell epitopes have also been predicted in the current study. B-cell based vaccines provide antibody-mediated immunity which can be easily overwhelmed by surge of antigens. However, HTL plays a crucial role in inducing vital humoral or CTL responses and confer long-term immunity; hence, critical requirements for effective vaccine design. The response to T-cell epitopes is restricted by HLA proteins. HLAs are highly polymorphic *i*.*e*. the frequency of expression of different HLA types varies in different ethnic human populations. Therefore, to elicit broad immune responses in different human populations, the HLA specificity of T-cell epitopes must be considered as major criteria for screening of the epitopes^56^. Consequently, the epitope candidate should bind to the maximum number of HLA alleles to get more population coverage. Hence, in this study 10 HTL epitopes that bind to the maximum number of HLA alleles were selected as putative HTL epitope candidates. The main adaptive immunity against bacteria was thought to be primarily humoral *i*.*e*. mediated by B-cell or CD4+ cell^8^. However, humoral immunity is not far enough to completely clean the infection, cell-mediated immunity is needed to induce cell death and completely destroy the bacterial habitat. Although pathogenic *Leptospira* is not considered as a typical intracellular pathogen, indeed some bacterial proteins may be able to escape from the phagolysosome and reach to the cytosol of host cells and are exposed to the host CD8+ T-cells response, as reviewed in ref.^57^. Though, recent studies have reported that cell-mediated immunity is involved in the protective immune response stimulated by the *Leptospira* pathogen or vaccines^58,59^. In the current study, one of the CD8+ restricted CTL epitope, ^619^ILVPGAWKY^627^ having high degree of conservancy among 46 serovars of pathogenic *Leptospira*, has also been predicted as antigenic site of linear B-cell and showing significant binding interaction with HLA-A*0201 protein; thus, considerably enhancing the possibility of this peptide to be a vaccine candidate. As per our knowledge, this immunoinformatics study represents novel vaccine candidates that will further aid in the development of improved vaccines for leptospirosis.

## Conclusion

Leptospirosis has emerged as a major concern globally and reasons for a large number of deaths in tropical regions of the world. Despite of that, the present therapeutic strategy available is very sporadic and unable to handle this alarming disease. The immunoinformatics based screening of vaccine target is a promising strategy to accelerate the vaccine development process and could conceivably be used as a cost-effective medical intervention for emerging infectious diseases. Our study starts with the identification of highly immunogenic and conserved outer membrane protein followed by the identification of B-cell, HTL and CTL epitopes. The vaccine candidates identified in the current study are highly conserved among 46 serovars of pathogenic *Leptospira*, and have not yet been assessed as vaccine candidates; and hence, could be worthy of further investigation as novel vaccine candidates. Furthermore, experimental studies will be required for immunogenicity testing, *in vitro* and in animal models to validate their efficacy as vaccine candidates against leptospirosis.

## Methods

### Protein sequence retrieval

Whole proteome of the LIC, encoding 3654 proteins was retrieved from the Universal Protein (UniProt) database (Proteome ID: UP000007037) (http://www.uniprot.org/proteomes/) in FASTA format and used for further analysis. UniProt is a comprehensive resource for protein sequences and annotation information which provide functional information about proteins with accuracy and consistency.

### Prediction of highest antigenic protein

Antigenicity refers to the ability of an antigen to induce the immune response. Hence, to find the highest antigenic protein, all protein sequences were submitted to VaxiJen v2.0 server (http://www.ddg-pharmfac.net/vaxijen/VaxiJen/VaxiJen.html) with default parameters, which was developed for the prediction of potent antigen and subunit vaccines with accuracy of 70 to 89%. All the antigenic proteins with highest antigenicity score (>1.0) were selected for further evaluation.

### Prediction of subcellular localization

It is important to scrutinize the subcellular localization of a protein, as immunogenic protein have to be easily recognized by the immune cells in order to stimulate immune response, one of the primary criteria for designing a vaccine candidate. Outer membrane proteins are surface-exposed which is easily recognised by the host immune system and possibly associated with pathogenesis^60^. Therefore, protein sequences with antigenic score >1.0 were subjected to CELLO v.2.5 server^61,62^ (http://cello.life.nctu.edu.tw/) to retrieve outer membrane protein.

### Homology modelling and structure analysis

Antigenicity or the function of a protein correlates with the structural features of the protein; hence, to analyse the target protein sequence, ProtParam server (http://web.expasy.org/protparam/) and SOPMA server (https://npsa-prabi.ibcp.fr/cgi-bin/npsa_automat.pl?page = /NPSA/npsa_sopma.html) were used with default parameters. ProtParam tool allows the computation of various parameters that decide the stability and functional characteristics of the protein to some extent and SOPMA computes the secondary structural features of the protein. The three-dimensional (3D) structure of outer membrane lipoprotein were predicted using the I-TASSER server^33,63^. I-TASSER generated five alternative 3D models of protein and assigned confidence score (C-score) for each model that infers the quality of the structure. The modelled protein with the highest C-score was refined and subjected for its quality assessment. The 3D model was refined by using GalaxyRefine server (http://galaxy.seoklab.org/). This server refines the modelled structure by reconstructing side-chain conformations followed by repacking and dynamics simulations to repeatedly relax the structure. GalaxyRefine has been evaluated as one of the best method to improve the local quality of the structure. This server can improve local and global quality of the models generated by structure prediction servers such as I-TASSER. Furthermore, to evaluate the refined model, quality assessment of the model was done by using three different servers *viz*. PROCHECK^64^, ProSA-Web^65,66^, and ModFold6^67^. PROCHECK was used to analyse the stereochemical quality of the model by evaluating the Ramachandran plot of the protein structure; whereas, ProSA-Web and ModFold evaluated the overall quality of the model. ProSA-Web calculated the overall quality score of the model by analysing their atomic coordinates which is frequently employed in protein tertiary structure validation. ModFold calculates the *p*-value and assign a degree of confidence (poor, low, medium, high and cert) of the model depending on the *p*-value. The 3D structure of protein was visualized using PyMol^68^.

### Linear and Conformational B-cell epitope prediction

B-cell epitope is the main antigenic region of an antigen which are recognized by the B-cell receptors of the immune system and are able to induce humoral immune response, which cause the B-lymphocytes to differentiate into antibody-secreting plasma and memory cells^69^. B-cell epitopes can be categorized as a linear (continuous) and conformational (discontinuous) based on their spatial structure. The Kolaskar & Tongaonkar method at Immune Epitope Database (IEDB) analysis resource (http://tools.iedb.org/main/bcell/) was applied to predict linear B-cell epitopes. The accuracy of this method to predict epitope is about 75%^35^. Flexibility, surface accessibility and hydrophilic properties are also important characteristics of B-cell epitopes^70^; hence, to predict these properties, Emini surface accessibility^36^, Karplus and Schulz Flexibility^37^ and Parker hydrophilicity^38^ prediction methods were employed respectively with default parameters of IEDB analysis resource.

ElliPro (http://tools.immuneepitope.org/toolsElliPro/) from IEDB analysis resource was used for prediction of the conformational B-cell epitopes with minimum score value set at 0.70, while the maximum distance was set as default. This method predicts epitopes based upon solvent-accessibility and flexibility^39^. Three different algorithms are implemented in this resource including approximation of the protein shape^71^, protrusion index (PI) of residues^72^ and neighbouring residues clustering based on their PI values.

### Helper T-cell (HTL) epitope prediction

Activation of HTL is prerequisite for inducing an efficient antibody response or Cytotoxic T-lymphocyte (CTL) response through both cytokine secretion and dendritic cell sensitization^73–75^. The binding of a T cell receptor to an epitope complexed with major histocompatibility complex (MHC) class II molecule can result in activation of T-cell. Hence, in order to predict MHC class II restricted HTL epitopes, the protein sequences were submitted to NetMHCIIpan 3.1 server (http://www.cbs.dtu.dk/services/NetMHCIIpan/) with threshold value set as 0.5% and 2% for strong binding peptides (SB) and weak binding peptides (WB), respectively to determine the binding affinities of epitopes and MHC-II allele. NetMHCIIpan is one of the most accurate prediction server that covers all human leucocyte antigen (HLA) class II molecules based on artificial neural network algorithm. Here, the strong binder epitopes with the maximum number of binding HLA-DR alleles were selected as putative epitope candidates.

### Prediction of Cytotoxic T-lymphocyte (CTL) epitopes

Consistent predictions of CTL epitopes are very important for the coherent vaccine design. Hence, the presence of CTL epitopes in the amino acid sequence of selected protein was predicted using NetCTL.1.2 server (http://www.cbs.dtu.dk/services/NetCTL), with default parameters. This server predicts epitopes by integrating predictions of MHC class I binding, proteasomal C-terminal cleavage and the TAP transport efficiency. The MHC class I binding and proteasomal C-terminal cleavage were predicted by the artificial neural network while a weight matrix was used to predict the TAP transport efficiency.

Moreover, except for a strong binding affinity, the peptides with strong immunogenicity are more probable CTL epitopes than those with weak immunogenicity. Therefore, the immunogenicity of candidate epitopes was evaluated using IEDB immunogenicity prediction tool (http://tools.immuneepitope.org/immunogenicity/) with default parameters.

### Allergenicity assesment

The allergenicity of the predicted epitopes was analysed using AllerHunter server (http://tiger.dbs.nus.edu.sg/AllerHunter), which is based on support vector machine (SVM) and pair-wise sequence similarity. AllerHunter predicts allergen in addition to non-allergen with high sensitivity and specificity, and efficiently distinguish allergens and non-allergens from allergen-like non-allergen sequences, which make AllerHunter a very constructive tool for allergen predictions.

### Conservancy analysis

In order to evaluate homologs of the selected proteins within different serovars of pathogenic *Leptospira* species, BLASTP (https://blast.ncbi.nlm.nih.gov/Blast.cgi?PAGE=Proteins) was performed against proteome of 47 serovars of pathogenic species. Protein sequences with >70% of identity and 40% query coverage were considered as homologs. Of these 47 serovars, the query protein was found to have their homologs among 46 serovars. Furthermore, conservancy of predicted epitope was evaluated among screened homologs (46 serovars) by using epitope conservancy analysis tool at the IEDB analysis resource (http://tools.immuneepitope.org/tools/conservancy). This tool calculates the degree of conservancy of an epitope within a provided protein sequence, set at different degree of identities. The degree of conservancy is defined as the portion of protein sequences that contain the epitope at a specified identity level.

### 3D structure of CTL-epitopes

The 3D structures of all the predicted CTL epitopes excluding the allergenic one *i*.*e*. SSSDLNLGI, were modelled with the PEP-FOLD3 server^76^, using 200 simulation runs. First the PEP-FOLD3 server clustered different conformational models and then sorted them using the sOPEP energy value. Consequently, the best ranked model was selected to analyse the interactions with selected Class I MHC molecule.

### Molecular Docking studies

A docking study was performed to ensure the interaction between HLA class I molecules and our predicted CTL epitopes using the PatchDock rigid-body docking server^77,78^. Since HLA-A*0201 is one of the most frequent MHC class I alleles in most of the human populations;^79–81^ the best ranked CTL peptide models were docked with HLA-A*0201 (PDB ID: 4U6Y). PatchDock rigid-body server computes complexes with good molecular shape complementarity based on geometry of the molecules. Furthermore, the docking results were refined using FireDock (Fast Interaction Refinement in Molecular Docking) server^82,83^. It produces 10 best solutions for final refinement. The refined models were based on the binding score. This score includes Atomic contact energy, Van Der Waals interaction, partial electrostatics and estimations of the binding energy. Furthermore, the hydrogen bonding interaction of the docked structures were analysed with the molecular visualization tool UCSF Chimera 1.11.2^84^ and PyMOL^68^.

### Molecular dynamics simulations

Molecular dynamics simulations were performed to check the stability of epitopes-HLA-A*0201 allele docked complex using the GROMACS v2016.3 software^85^. For each of the docked complexes, a production simulation of 5 ns at 300 K temperature and 1 bar pressure was obtained after carrying out stepwise energy minimization and equilibration protocol of the solvated systems with TIP3P water model. Further, trajectory analysis was performed to investigate H-bonding and Root Mean Square Deviation (RMSD).

## Electronic supplementary material

Supplementary Dataset

Supporting Information
