# Supplementary material for: Exploring Leptospiral proteomes to identify potential candidates for vaccine design against Leptospirosis using an immunoinformatics approach
Source: Sci Rep. 2018 May 2;8:6935. doi: 10.1038/s41598-018-25281-3 (PMC5932004; doi:10.1038/s41598-018-25281-3)
Supplement: Supplementary file 2 — Supporting Information [file 41598_2018_25281_MOESM2_ESM.pdf]

Supporting Information

Exploring Leptospiral proteomes to identify potential candidates for vaccine design against Leptospirosis using an immunoinformatics approach

Kumari Snehkant Lata[1, 3], Swapnil Kumar[1, 3], Vibhisha Vaghasia[1, 3], Priyanka Sharma[1, 3], Shivarudrappa B. Bhairappanvar[1, 3], Subhash Soni[2, 3] & Jayashankar Das[2, 3, *]

[1] Gujarat Institute of Bioinformatics, Gujarat State Biotechnology Mission, Department of Science & Technology, Government of Gujarat, Gandhinagar – 382011, India.

[2] Gujarat State Biotechnology Mission, Department of Science & Technology, Government of Gujarat, Gandhinagar – 382011, India.

[3] Present Address: Gujarat Biotechnology Research Centre (GBRC), Department of Science & Technology, Government of Gujarat, Gandhinagar – 382011, India.

*** Corresponding author. Tel: +91 (079)23252166; E-mail: jd2-gbrc@gujarat.gov.in, jayshankardas@gmail.com**

Table S2. Subcellular localization of the 21 highly antigenic proteins (antigenic score >1) of LIC

			Subcellular localization				
**UniProt ID**	**Gene**	**Gene product**	**Extracellular**	**Periplasmic**	**Cytoplasmic**	**Inner Membrane**	**Outer Membrane**
Q72M98	LIC_13290	Uncharacterized protein	✓	-	-	-	-
Q72SR0	LIC_11315	Uncharacterized protein	-	-	-	✓	-
Q72QN0	LIC_12083	Uncharacterized protein	-	-	✓	-	-
Q72VS9	LIC_10217	Uncharacterized protein	-	-	-	✓	-
Q72PW1	LIC_12356	Uncharacterized protein	-	-	✓	-	-
Q72NL5	LIC_12819	Uncharacterized protein	-	-	-	✓	-
Q72UU1	LIC_10566	Uncharacterized protein	-	✓	-	-	-
Q72PD8	LIC_12533	Uncharacterized protein	-	-	✓	-	-
Q72NS5	LIC_12754	Uncharacterized protein	-	-	-	✓	-
Q72PG9	LIC_12502	Putative sigma-54 modulation protein	-	-	✓	-	-
Q72RA2	LIC_11846	Flagellar motor switch protein FliN	✓	-	-	-	-
Q72N98	LIC_12938	Uncharacterized protein	✓	-	-	-	-
Q72NY0	LIC_12698	Uncharacterized protein	-	-	-	✓	-
Q72VZ0	rpmH	50S ribomal protein L34	-	-	✓	-	-
Q72NQ6	rpmA	50S ribomal protein L27	-	-	✓	-	-
Q72R35	LIC_11916	Putative lipoprotein	✓	-	-	-	-

Q72SG0	LIC_11423	Glycine rich RNA-binding protein	✓	-	-	-	-
Q75FL0	LIC_20172	Lipoprotein	-	-	-	-	✓
Q72PD2	LIC_12539	Uncharacterized protein	-	-	-	-	✓
Q72LR6	LIC_13487	Cytoplasmic membrane protein	-	-	-	✓	-
Q72RK1	LIC_11744	Uncharacterized protein	-	-	✓	-	-

Table S4: CTL epitopes and HLA-A*0201 interactions

Peptide	Global energy (kcal/mol)[a]	Attractive vdW energy (kcal/mol)	H-bond energy (kcal/mol)	H-bond interaction		
				Epitope-MHC atom pair[b]	Distance initial[c] (Å)	Distance after MD[d] (Å)
NSDSSSNAT	-20.19	-24.41	-3.28	SER 6 OG-GLN 32 OE1	2.94	nd
				SER 4 OG-GLY 237 O	2.20	nd
				ALA 8 O-THR 31 OG1	3.03	nd
				SER 6 O-GLN 32 N	2.02	nd
GTSYKDWYK	-30.68	-18.36	-2.74	LYS 9 O1-ARG 65 NE	3.56	nd
				TRP 7 O-ARG 65 HH22	nd	2.07
				GLY 1 H1-GLU 58 OE1	nd	2.25
				GLY 1 H1-GLU 58 OE2	nd	1.77
				LYS 9 O-TRP 167 HE1	nd	1.87
VSDNEGHIL	-38.80	-25.13	-2.14	HIS 7 ND1- THR 73 OG1	2.11	nd
				GLU 5 O-THR 73 OG1	3.98	nd
				GLY 6 O-ARG 97 HH22	nd	2.28
				GLU 5 O-THR 73 HG1	nd	1.79
				ASP 3 O-HIS 70 HE2	nd	1.88
				ASN 4 OD1-ARG 65 HH21	nd	1.64
				ASN 4 OD1-ARG 65 HE	nd	2.25

				GLY 6 O-ARG 97 HH12	nd	3.31
YSSSFILII	-39.30	-23.37	-1.28	ILE 6 O-LYS 66 NZ	2.09	nd
				SER 4 OG-GLU 58 OE2	3.00	nd
				TYR 1 OH-THR 163 O	3.83	nd
				LEU 7 O-THR 163 OG1	4.03	nd
				ILE 9 O-ARG 97 HH22	nd	1.75
VTDLTTKTV	-15.27	-23.06	-3.45	THR 2 O-THR 73 OG1	2.73	nd
				THR 5 OG1-TYR 99 OH	3.50	nd
				VAL 1 H1-ASP 77 OD1	nd	1.71
				ASP 3 OD1-ARG 97 HH22	nd	2.12
				ASP 3 OD2-ARG 97 HH22	nd	1.83
				ASP 3 OD2-ARG 97 HH12	nd	1.67
YLDSNNFPW	-34.41	-29.62	-3.64	SER 4 OG-LYS 146 NZ	2.63	nd
				ASN 5 O-TYR 116 OH	2.93	nd
				PHE 7 O-THR 73 OG1	1.99	nd
				ASN 6 O-ARG 97 NH1	3.62	nd
				TYR 1 OH-LEU 156 O	3.73	nd
				ASN 6 O-ARG 97 NH2	3.95	nd
WVASNGTSY	-28.08	-28.85	-2.99	ASN 5 ND2-TYR 113 OH	3.21	nd

				ASN 5 OD1-ARG 6 NH2	3.48	nd
				TYR 9 OH-SER 2 OG	2.87	nd
				TYR 9 N-GLU 264 OE1	2.97	nd
				TRP 1 N-SER 2 O	2.64	nd
				SER 8 OG-GLU 212 O	3.66	nd
				TRP 1 N-GLU 264 OE1	4.03	nd
				TRP 1 HE1-THR 182 OG1	nd	2.18
KTALGSYPY	-61.00	-28.32	-3.18	TYR 9 OH-GLU 232 OE1	2.42	nd
				SER 6 OG-SER 4 OG	3.32	nd
				LEU 4 N-GLU 212 OE1	3.85	nd
				SER 6 HN-ASP 30 OD2	nd	2.86
				TYR 7 HH-GLU 232 OE2	nd	1.78
				LYS 1 H3-GLU 212 OE2	nd	1.71
				TYR 7 O-TYR 27 HH	nd	1.86
				SER 6 HG1-ASP 29 O	nd	2.02
				LYS 1 HZ1-ASP 29 OD1	nd	1.67

[a]FireDock energy for the best ranked complex. [b]Interacting pair of atoms and residues between epitope and HLA-A*0201. [c]Initial distance between the H-bond acceptor and the donor. [d]Distance between the H-bond acceptor and the donor after the MD simulation. nd = H-bond not detected.

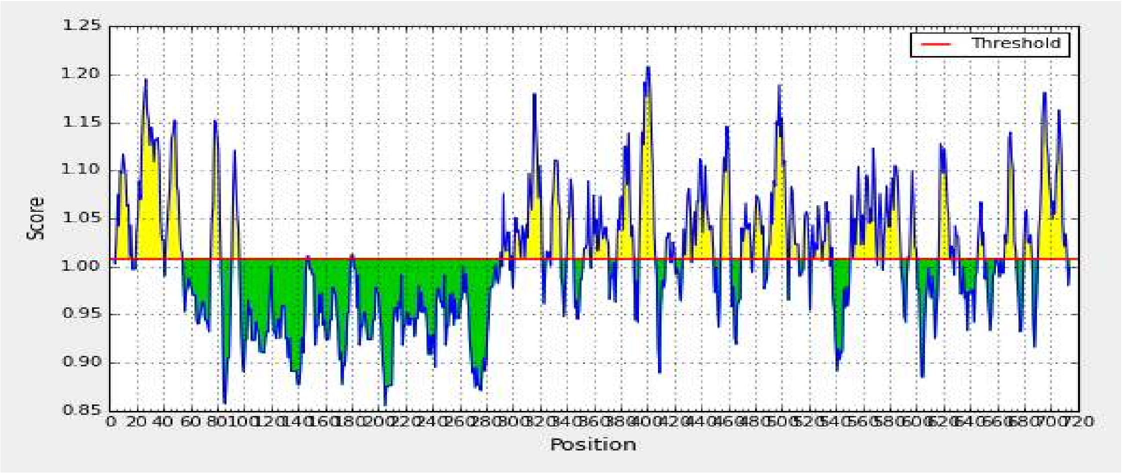

Fig. S1. Graphical representation of the predicted antigenic propensity of lipoprotein Q75FL0

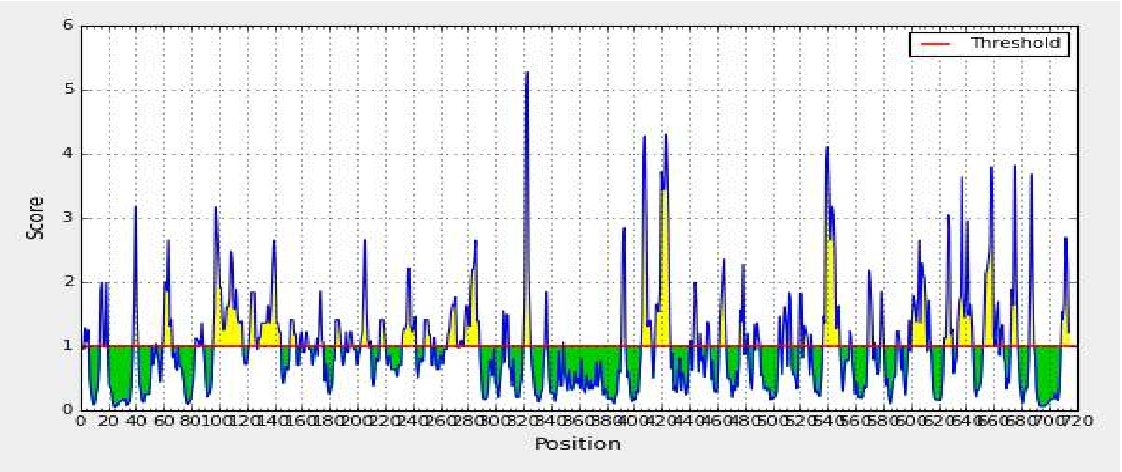

Fig. S2. Graphical representation of the predicted surface probability of lipoprotein Q75FL0

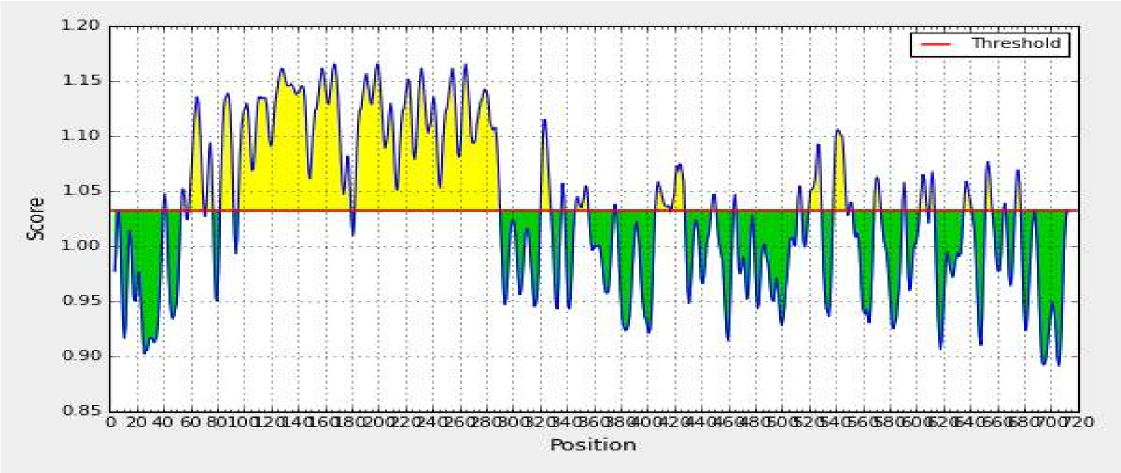

Fig. S3. Graphical representation of the predicted surface flexibility of lipoprotein Q75FL0

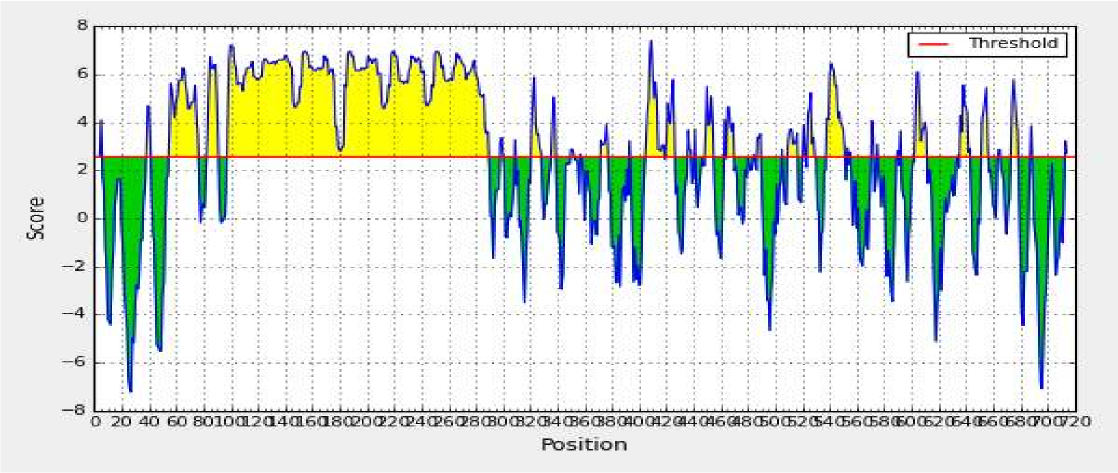

Fig. S4. Graphical representation of the predicted surface hydrophilicity of lipoprotein Q75FL0

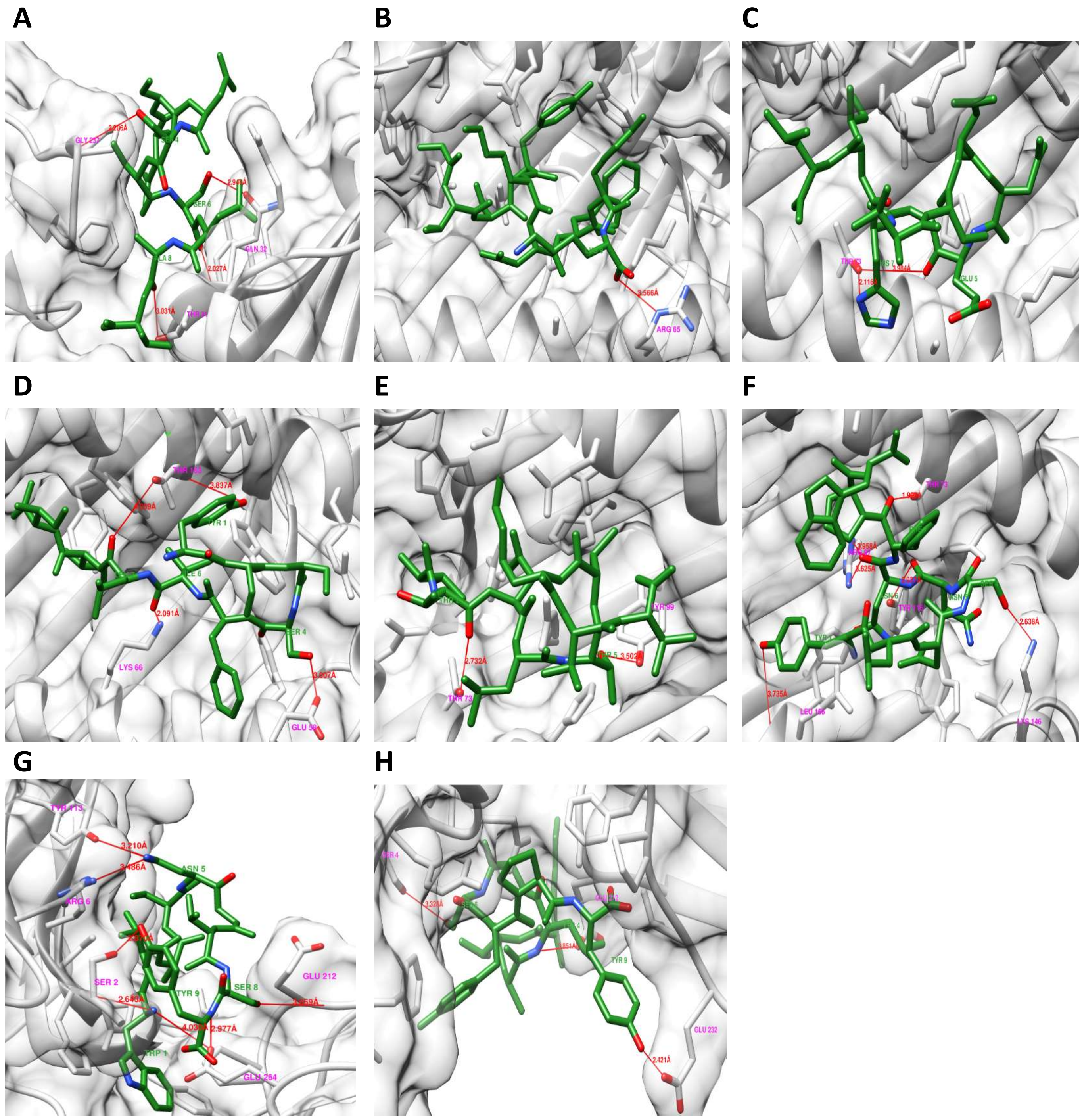

Fig. S5: Molecular interaction analysis of the predicted CTL epitopes docked to MHC-I HLA-A*0201 protein. (A) NSDSSSNAT; (B) GTSYKDWYK; (C) VSDNEGHIL; (D) YSSSFILII; (E) VTDLTTKTV; (F) YLDSNNFPW; (G) WVASNGTSY; (H) KTALGSYPY. The residues forming H-bonds are labelled in magenta and green colour for HLA protein and epitope respectively. Each residue participating in H-bonding are coloured with Atom type (Red: Oxygen, Blue: Nitrogen).

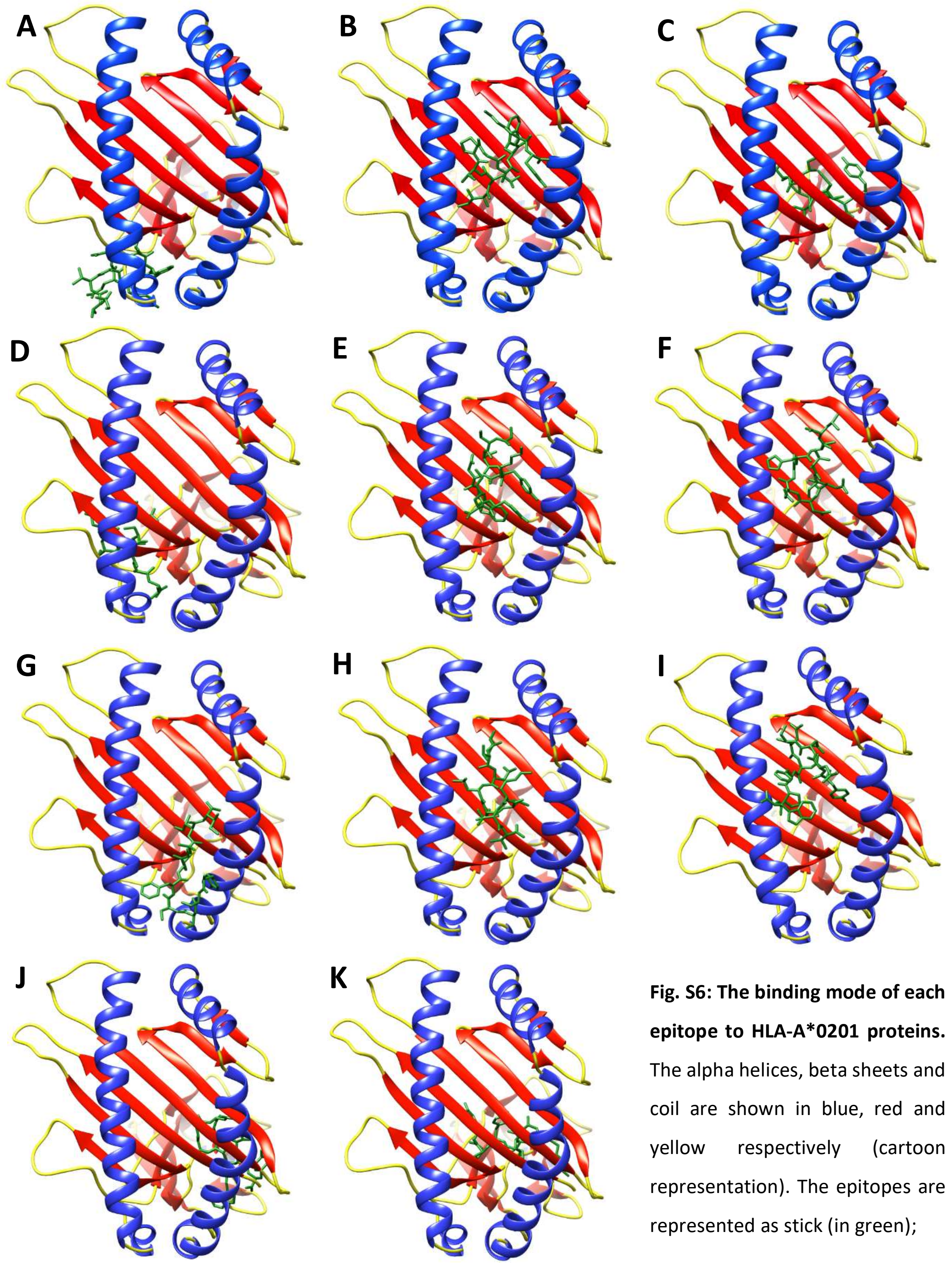

Fig. S6: The binding mode of each epitope to HLA-A*0201 proteins. The alpha helices, beta sheets and coil are shown in blue, red and yellow respectively (cartoon representation). The epitopes are represented as stick (in green);

(A) STVAYEDLY-HLA-A*0201; (B) ILVPGAWKY-HLA-A*0201; (C) QIGSIPFTY-HLA-A*0201; (D) NSDSSSNAT-HLA-A*0201; (E) GTSYKDWYK-HLA-A*0201; (F) VSDNELA-A*0201;(G) YSSSFILII-HLA-A*0201; (H) VTDLTTKTV-HLA-A*0201; (I) YLDSNNFPW-HLAA*0201; (J) WVASNGTSY-HLA-A*0201; (K) KTALGSYPY- HLA-A*0201.

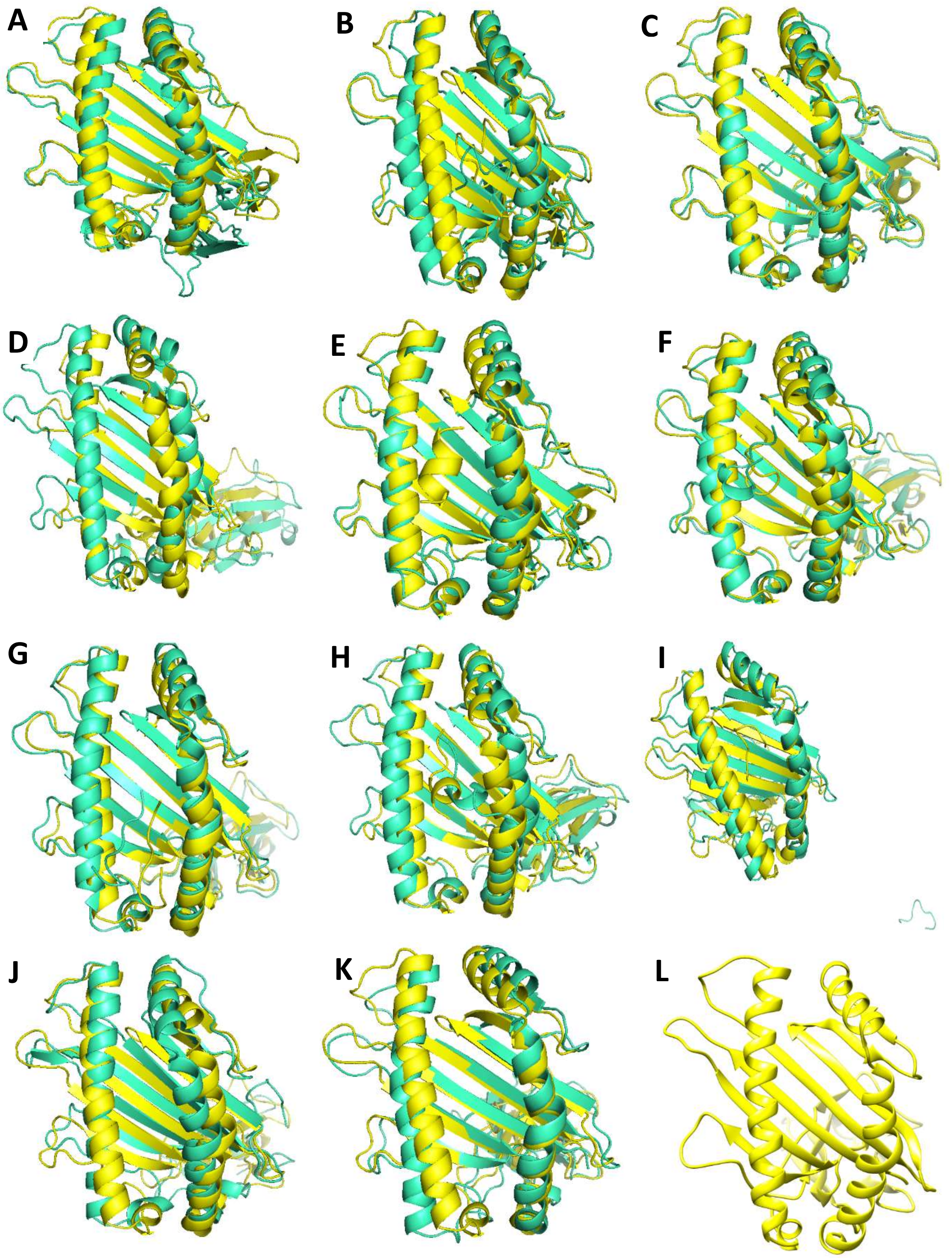

Fig. S7: CTL epitopes- HLA-A*0201 complexes (cartoon representation). The original docked complex (in yellow) is superimposed with the complex after MD simulation (in greencyan): (A) STVAYEDLY; (B) ILVPGAWKY; (C) QIGSIPFTY; (D) NSDSSSNAT; (E) GTSYKDWYK; (F) VSDNEGHIL; (G) YSSSFILII; (H) VTDLTTKTV; (I) YLDSNNFPW; (J) WVASNGTSY; (K) KTALGSYPY; (L) Front view of the HLA-A*0201 binding groove.
